# Perthe’s Disease

**Published:** 1918-08

**Authors:** 


**Perthe's Disease.** C. B. Francisco of Kansas City, Med. Herald, Jan. 1918, reviews the literature brieflly. The disease is a benign deforming osteochondritis, due to delayed ossification of the epiphysis and juxta-ephiphyseal cartilage of the neck and an isolated area of the acetabulum, characterized by slight limp and pain. 80% of cases occur in boys between 5 and 10. Diagnosis is established by Roentgenology. Extension and massage, without fixation are the means advised.

